# A simple but tough-to-beat baseline for fMRI time-series classification

**DOI:** 10.1016/j.neuroimage.2024.120909

**Published:** 2024-11-06

**Authors:** Pavel Popov, Usman Mahmood, Zening Fu, Carl Yang, Vince Calhoun, Sergey Plis

**Affiliations:** a Tri-institutional Center for Translational Research in Neuroimaging and Data Science (TReNDS), Georgia State University, Georgia Institute of Technology, Emory University, Atlanta, 30303, GA, USA; b Georgia State University, Atlanta, 30303, GA, USA; c Emory University, Atlanta, 30303, GA, USA

**Keywords:** Resting-state fMRI, Data explainability, Machine learning, Deep learning, Brain disorders, Predictive neuroimaging

## Abstract

Current neuroimaging studies frequently use complex machine learning models to classify human fMRI data, distinguishing healthy and disordered brains, often to validate new methods or enhance prediction accuracy. Yet, where prediction accuracy is a concern, our results suggest that precision in prediction does not always require such sophistication. When a classifier as simple as logistic regression is applied to feature-engineered fMRI data, it can match or even outperform more sophisticated recent models. Classification of the raw time series fMRI data generally benefits from complex parameter-rich models. However, this complexity often pushes them into the class of black-box models. Yet, we found that a relatively simple model can consistently outperform much more complex classifiers in both accuracy and speed. This model applies the same multi-layer perceptron repeatedly across time and averages the results. Thus, the complexity and black-box nature of the parameter rich models, often perceived as a necessary trade-off for higher performance, do not invariably yield superior results on fMRI.

Given the success of straightforward approaches, we challenge the merit of research that concentrates solely on complex model development driven by classification. Instead, we advocate for increased focus on designing models that prioritize the explainability of fMRI data or pursue applicable objectives beyond mere classification accuracy, unless they significantly outperform logistic regression or our proposed model. To validate our claim, we explore possible reasons for the superior performance of our straightforward model by examining the innate characteristics of fMRI time series data. Our findings suggest that the sequential information hidden in the temporal order may be far less important for the accurate fMRI classification than the stand-alone pieces of information scattered across the frames of the time series.

## 1. Introduction

Novel approaches for analyzing human brain fMRI data are rapidly being developed. Often their aim is to deepen our understanding of the inner workings of the human brain with the aim of identifying biomarkers of brain disorders. Machine learning (ML) techniques have been widely used to improve diagnostic sensitivity in a range of disorders including (Liu et al., 2021a; Bondi et al., 2023; de Filippis et al., 2019; Warren and Moustafa, 2023), or to predict an individuals sex and age (Yeung et al., 2023) or behavioral assessment. However, advanced approaches often suffer from a lack of fMRI data explainability due to difficulties associated with both ML models interpretability and the high dimensionality and low signal to noise ratio of the data itself. Current ML models working with the brain fMRI data have been used to analyze time series data as well as functional connectivity. Time series fMRI captures the dynamics of blood-oxygenation-level-dependent (BOLD) signals in the brain (Kundu et al., 2017), which correlate with the brain activity. While fMRI images are captured at the voxel level in the 3D space, they are often summarized (parcellated) by averaging the signals within brain regions or networks. These regions can be derived either from an anatomical atlas (Desikan et al., 2006; Schaefer et al., 2017) (region of interest (ROI) parcellation), resulting in separate, typically non-overlapping parcels or from the data itself, e.g., by using independent component analysis (Fu et al., 2019) (ICA parcellation) resulting in overlapping whole brain networks. Functional network connectivity (FNC) captures the correlations of activity between brain regions (van den Heuvel and Hulshoff Pol, 2010); it is typically derived by computing Pearson cross-correlation matrices from the fMRI time series.

Historically, the majority of models for fMRI data were designed to work with the FNC data (Khosla et al., 2019; Rish et al., 2009; Shen et al., 2010; Arbabshirani et al., 2013). Some of the earlier attempts to utilize the ML models for brain disorders classification were applying classic techniques, such as logistic regression (Cox, 1958), support vector machines (Boser et al., 1992), naïve bayes, or k-nearest neighbor (Fix and Hodges, 1989), to the FNC data computed either from the voxel-level or parcellated fMRI time series. These approaches typically made conclusions about the links between brain function and brain disorders via the analysis of the most discriminative features in the data. More recently, deep learning techniques such as convolutional neural networks (CNNs) (Kawahara et al., 2017), graph neural networks (Kan et al., 2022a), and transformer (Kan et al., 2022b) modules, have been used to take into account additional prior knowledge about the brain, such as topological relations between the FNC components (Kawahara et al., 2017; Bannadabhavi et al., 2023). However the new models have similar, or even greater challenges with providing interpretable results.

At the same time, the advances of deep learning techniques allowed the development of models that work with fMRI time series. Some recent models have even made attempts to work directly with the volume unparcellated fMRI time series (Huang et al., 2021; Malkiel et al., 2022; Kim et al., 2023). In line with the latest trends in machine learning, Caro et al. (2023) recently introduced a huge foundation model for fMRI analysis. Many models also focus on directly learning the functional relationship in fMRI time series with a flexible model rather than using Pearson correlation. This idea is illustrated in dynamics of recent models of fMRI time series that focus on learning the effective connectivity matrices rather than rigidly estimating them (Mahmood et al., 2021; Kan et al., 2022a; Mahmood et al., 2022, 2023). In these cases, the effective FNC can be learned for a specific classification task; potentially providing a more interpretable and discriminative functional connectivity profile. However, in this paper we show that logistic regression trained on statistically derived FNC data results in classification performance comparable to or surpassing that of more complex and recent models. In this context, working with the fMRI time series appears to be more challenging in terms of model architecture design and rewarding for data explainability.

In this work we present a relatively simple model based on multi-layer perceptron (MLP) for classification on the fMRI time series that we call meanMLP. We show in extensive comparisons that our model is capable of accurate fMRI classification. More interestingly, our model’s performance is comparable, and often even superior, to that of much more intricate models for fMRI time series in terms of both accuracy and speed. The use of the MLP-only architecture was initially inspired by the recent spark in interest to the models with little inductive bias, i.e. the models with less restrictive characteristics embedded in their architecture. As such, in the recent works the MLP-only architectures were tested on the vision problem (Tolstikhin et al., 2021; Bachmann et al., 2023) and found to be quite efficient compared to more conventional CNN and Transformer architectures. In this context, meanMLP represents an MLP-only architecture for the time series classification, which can be viewed as a trivialized version of RNN architecture with no information flow between the input time points. The classification success and simplicity of our model puts it in a close reference to the linear models for time forecasting recently explored by Zeng et al. (2022).

Considering the classification success of our simplistic approach, we believe that a greater emphasis in the future ML research in application to neuroimaging should be put on matters beyond classification accuracy, e.g., the problem of fMRI data explainability. To support this idea, and to find an explanation for our model’s success, we explored the properties of the fMRI time series. By analyzing the influence of different preprocessing techniques and time-shuffling on the models’ performance, we provide empirical evidence that the dynamical information embedded in the temporal order may be far less important for an accurate fMRI classification than it is commonly believed, and sufficiently discriminative features in the data might be simply dispersed across the frames of the time series.

## 2. Methods

In this section we introduce our baseline model that turned out surprisingly strong, the other models we used in our experiments for comparisons, the fMRI datasets and their preprocessing pipeline, and the experiment designs we used to evaluate and analyze our models and the data they work with.

### 2.1. Models

In our work we benchmarked a total of 11 models, 7 of which were specifically designed for classification on fMRI data (Mahmood et al., 2020, 2022; Kawahara et al., 2017; Kan et al., 2022a,b; Bedel et al., 2023; Mahmood et al., 2023). Here we will first briefly review the models that work with fMRI time series, starting with introducing our model, and then move to the models that work with the FNC input.

#### 2.1.1. meanMLP model

**Fig. 1.**
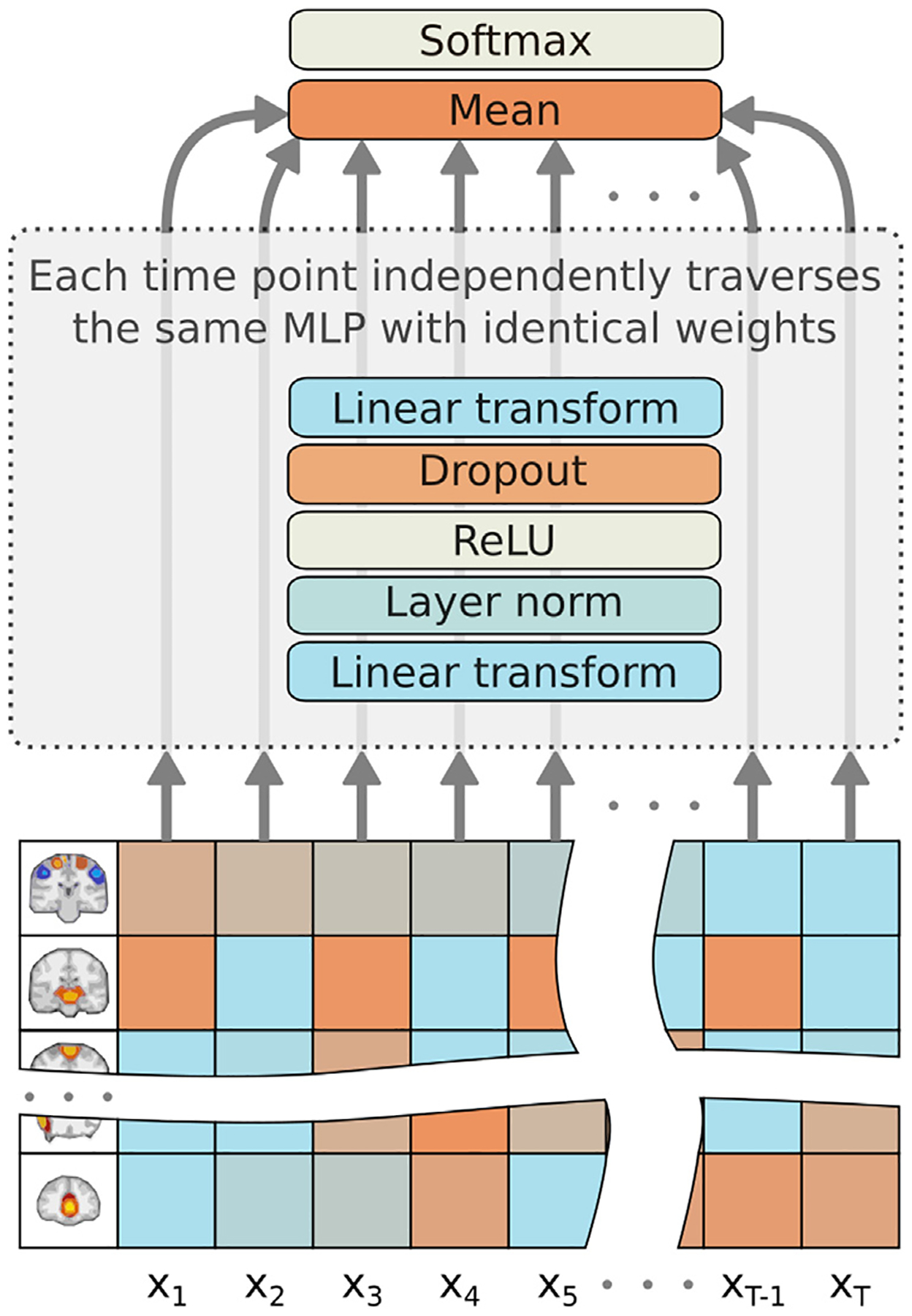
Schematic view of the forward pass of the meanMLP model. Note: different time points in the input data propagate through the same MLP block with the same weights, what is often referred to as parameter tying.

A schematic view of the meanMLP model is shown in Fig. 1. meanMLP model consists of a notably simple two layer MLP, with dropout rate and hidden state size *d* as hyperparameters. To describe the model more rigorously, let a single fMRI time series sample be $\left\{x,y\right\},x\in {\mathbb{R}}^{T\times k},y\in {\mathbb{R}}^{C}$, where *T* is the number of time points, *k* is the input feature size (#ROIs/#ICs), and *C* is the number of classes. In the meanMLP model, each time point $x_{t}\in {\mathbb{R}}^{k},t\in \left\{1,2,\ldots ,T\right\}$ is processed by the same MLP independently. The following set of equations summarizes the model’s forward propagation:

$$a_{t}^{\left(0\right)}=\text{Dropout}\left(\text{ReLU}\left(\text{Layer}\;\text{Norm}\left(W^{\left(0\right)}x_{t}+b^{\left(0\right)}\right)\right)\right),\;W^{\left(0\right)}\in {\mathbb{R}}^{d\times k},b^{\left(0\right)}\in {\mathbb{R}}^{d};\;h_{t}^{\left(1\right)}=W^{\left(1\right)}a_{t}^{\left(0\right)}+b^{\left(1\right)},\;W^{\left(1\right)}\in {\mathbb{R}}^{C\times d},b^{\left(1\right)}\in {\mathbb{R}}^{C};\;h_{\text{mean}}=\frac{1}{T}\;\overset{T}{\underset{t=1}{\sum }}h_{t}^{\left(1\right)};\;\hat{y}=\text{Softmax}\left(h_{\text{mean}}\right).$$

Here $\hat{y}\in {\mathbb{R}}^{C}$ is the model’s prediction for the sample *x*.

We notice that the *mean* operation used to calculate *h*_mean_ makes the model permutation invariant on and thus insensitive to the time order in the data; it is possible to reshuffle the data along the time axis with no effect on model’s output. Also, the *mean* operation likely allows the model to even out the noise in the otherwise fairly noisy fMRI time series; a similar technique was used before by Dvornek et al. (2017) in an LSTM design for fMRI classification.

#### 2.1.2. Existing models for fMRI time series

Last decade advancements in deep learning have led to the development of various models for brain fMRI data that utilize RNNs, CNNs, and Transformer modules (Valliani et al., 2019). In our work we use a few of such models to compare the performance of meanMLP model.

**Long Short-Term Memory** (LSTM) is a long-history recurrent neural network widely used for the sequence data, such as time series (Hochreiter and Schmidhuber, 1997). In our work we use it to compare the meanMLP model with some relatively simple and general models. In our implementation of an LSTM classifier we used an LSTM block (Hochreiter and Schmidhuber, 1997) followed by a fully connected (FC) layer that performed classification on the last output embedding of LSTM block, or concatenated first and last embedding in bidirectional LSTMs (bidirectionality was treated as a hyperparameter).

meanLSTM, a modification of the LSTM model, follows the same design, with the exception of using a mean output LSTM embedding for classification. Such averaging is supposed to bridge meanMLP and LSTM models, placing meanLSTM and its expected behavior somewhere in between these two. We note that this design is more similar to the LSTM design for fMRI classification presented in Dvornek et al. (2017), which may be more known in the neuroimaging society compared to the more traditional design above.

**Transformer** model, another general architecture we used in model comparisons, received a significant attention in the last decade that uses self-attention mechanism (Vaswani et al., 2017). In our implementation we used a BERT-like transformer encoder architecture (Devlin et al., 2019) for classification on fMRI time series. We used a transformer encoder, preceded by an FC layer and ReLU that transformed the input fMRI features at each time point to the input embeddings of the encoder, and a sine-wave positional encoding block. On top of the encoder we added an FC layer for classification on the first output encoder embedding.

Similarly to the meanLSTM model, meanTransformer mimics the Transformer model architecture, with the exception of using the mean output encoder embedding for classification.

**Mutual information local to context** (MILC) model introduced in Mahmood et al. (2020) is a classification-focused model for fMRI time series that uses CNN modules. MILC utilizes a 1D CNN encoder to extract representations of time windows obtained by sliding a window of fixed length across time of fMRI time series. The outputs of the CNN are passed to a bidirectional LSTM, followed by an attention module that assigns weights for the LSTM outputs. A weighted sum of LSTM outputs is then used in classification. An interesting feature of the MILC model is that it allows for pre-training of the CNN encoder on unrelated fMRI data, that helps to improve the overall model performance.

**Directed Instantaneous Connectivity Estimator** (DICE) (Mahmood et al., 2022), another model for the fMRI time series, takes a different approach by focusing on not only the classification performance, but also the data explainability. In DICE, the each features’ time series is passed through a bidirectional LSTM to infer the feature’s embedding at each time point. These embeddings are then passed through a self-attention mechanism to retrieve the spatial relations between embeddings at each time point. Finally, the DICE model utilizes a global temporal attention module to derive a task-specific global directed network connectivity (DNC), which is more task-related compared to statistically derived FNC matrices, and allows for better interpretablility. Besides that, global DNC is also used in classification.

**Glacier**, a transformer-based model (Mahmood et al., 2023), implements an approach quite similar to DICE, both in goals and design. However, where DICE utilizes LSTM for deriving latent states of brain regions at each time point, Glacier uses a transformer encoder. In the end, this model also estimates a global DNC matrix that shows more task-related interactions.

**BOLD Transformer** (BolT) is another transformer-based model for fMRI time series (Bedel et al., 2023). Unlike vanilla transformers that process the whole time series globally, BolT implements a hierarchical approach by splitting the time series into overlapping windows and passing them through cascade of transformers. Apart from better efficiency at processing long time series, such approach allows it to derive features in a local-to-global manner by fusing and transforming tokens from neighboring windows until a global token is derived. This global token then can be used both for classification and as a reference for detection of the most predictive time points, which can be further investigated to derive explanations on data.

**SwiFT**, a Swin transformer-based model (Kim et al., 2023), is another type of hierarchical transformer developed for volume (unparcellated) fMRI time series. SwiFT extends the architecture of sliding window visual transformers (Liu et al., 2021b) to process 4D fMRI data. Similar to BolT, its hierarchical structure is designed to capture longer sequential features more effectively. Additionally, by working with unparcellated fMRI data, SwiFT has the potential to capture finer spatio-temporal features, which are unavailable to models working with parcellated data.

#### 2.1.3. FNC models

The rest of the models we used in our comparisons utilize FNC fMRI data, either exclusively or as an addition to the time series input.

**BrainNetCNN** (Kawahara et al., 2017) is a classification-focused model for the FNC fMRI input that uses CNN modules. While originally it was designed for diffusion tensor imaging (DTI) data, it can be seamlessly adapted for the FNC fMRI input. BrainNetCNN uses special-shaped CNN patches that are designed for the brain network connectivity data. Edgeto-edge layers use cross-shaped patches on the FNC matrix to produce several channels of refined node connections. Edge-to-node layers use strip patches to produce a vector of node outputs. Node-to-graph layers use strip patches to produce a single generalized node output. The final CNN output is used for classification.

**Functional Brain Network Generation** (FBNetGen) model (Kan et al., 2022a) takes an approach very similar to DICE by deriving DNC matrices from the fMRI time series input. In FBNetGen, each feature’s time series is split into adjacent windows and passed through a bidirectional GRU. The softmax of the final GRU output is interpreted as a global feature embedding. The DNC matrix is then derived as an outer product of feature embeddings. However, unlike DICE, FBNetGen uses the derived DNC *along with* the FNC data to provide the final prediction by passing them to a graph convolution network-based predictor (Kipf and Welling, 2016). For this reason we include FBNetGen in the FNC model category.

**Brain Network Transformer** (Kan et al., 2022b), further referred to as BNT, models the brain networks as a graph, treating the correlation profiles from the FNC matrices as node embeddings and brain regions as nodes. BNT utilizes a multi-layer multi-head self-attention module typical for transformers (Vaswani et al., 2017) to compute the enhanced network connectivity from the FNC fMRI input. It then compresses the enhanced nodes into graph embeddings by clustering functionally similar nodes using an orthonormal clustering readout (OCRead), a graph readout function designed by the authors of BNT specifically for the fMRI data. The BNT has been shown to outperform various alternative models of different architectural types, including SAN (Kreuzer et al., 2021), Graphomer (Ying et al., 2021), BrainGNN (Li et al., 2021), BrainGB (Cui et al., 2023), BrainNetCNN, FBNetGEN, and DGM (Kazi et al., 2023).

**Logistic regression** (LR) fills the niche of a simpler and general use model that we used as a baseline for other FNC models. In our work we employed a scikit-learn implementation (Pedregosa et al., 2011) with default hyperparameters, using flattened upper FNC triangles as input.

To summarize, in our work we used 8 classification model for fMRI time series input:

- **meanMLP**, our proposed hard-to-beat baseline;
- **LSTM**, a general model for sequences;
- **Transformer**, another general model for sequences;
- **MILC**, a classification-focused model designed for fMRI;
- **DICE**, an interpretability-focused model for fMRI that learns directed connectivity;
- **Glacier**, another interpretability-focused model for fMRI that learns directed connectivity;
- **BolT**, an hierarchical transformer model for fMRI that can reveal most discriminative time points;
- **SwiFT**, another hierarchical transformer model that works directly with the volume fMRI time series;

and 4 models for FNC fMRI input:

- **BrainNetCNN**, a classification-focused model designed for DTI data, but adaptable for FNC fMRI;
- **FBNetGen**, an interpretability-focused model that derives DNC from the fMRI time series and uses it with FNC fMRI for classification;
- **BNT**, a transformer/graph model for FNC fMRI with some interpretability in mind;
- **LR**, a simpler general model used as a baseline for other FNC models.

### 2.2. Datasets

We used resting-state fMRI images collected from FBIRN (Function Biomedical Informatics Research Network) (Keator et al., 2016), COBRE (Center of Biomedical Research Excellence) (Çetin et al., 2014), BSNIP (Bipolar and Schizophrenia Network for Intermediate Phenotypes) (Tamminga et al., 2014), ABIDE (Autism Brain Imaging Data Exchange, release 1.0) (Di Martino et al., 2014), OASIS (Open Access Series of Imaging Studies, release 3.0) (Rubin et al., 1998), ADNI (Alzheimer’s Disease Neuroimaging Initiative) (Petersen et al., 2010), HCP (Human Connectome Project, 1200 subjects release) (Van Essen et al., 2013), and UK Biobank.^1^ Information about these datasets is shown in Table 1.

#### 2.2.1. Preprocessing pipeline

All images from the datasets other than HCP and UKB were preprocessed using statistical parametric mapping (SPM12^2^) under MATLAB 2022 environment. A rigid body motion correction was performed using the toolbox in SPM to correct subject head motion, followed by the slice-timing correction to account for timing difference in slice acquisition. The fMRI data were subsequently warped into the standard Montreal Neurological Institute (MNI) space using an echo planar imaging (EPI) template and were slightly resampled to 3 × 3 × 3 mm^3^ isotropic voxels. The resampled fMRI images were finally smoothed using a Gaussian kernel with a full width at half maximum (FWHM) = 6 mm.

For the images coming from UKB and HCP datasets, we used the minimally preprocessed data from the repository prepared according to Glasser et al. (2013). Like the rest of the datasets, we normalized them into the MNI space and smoothed with a 6 mm Gaussian kernel.

**Fig. 2.**
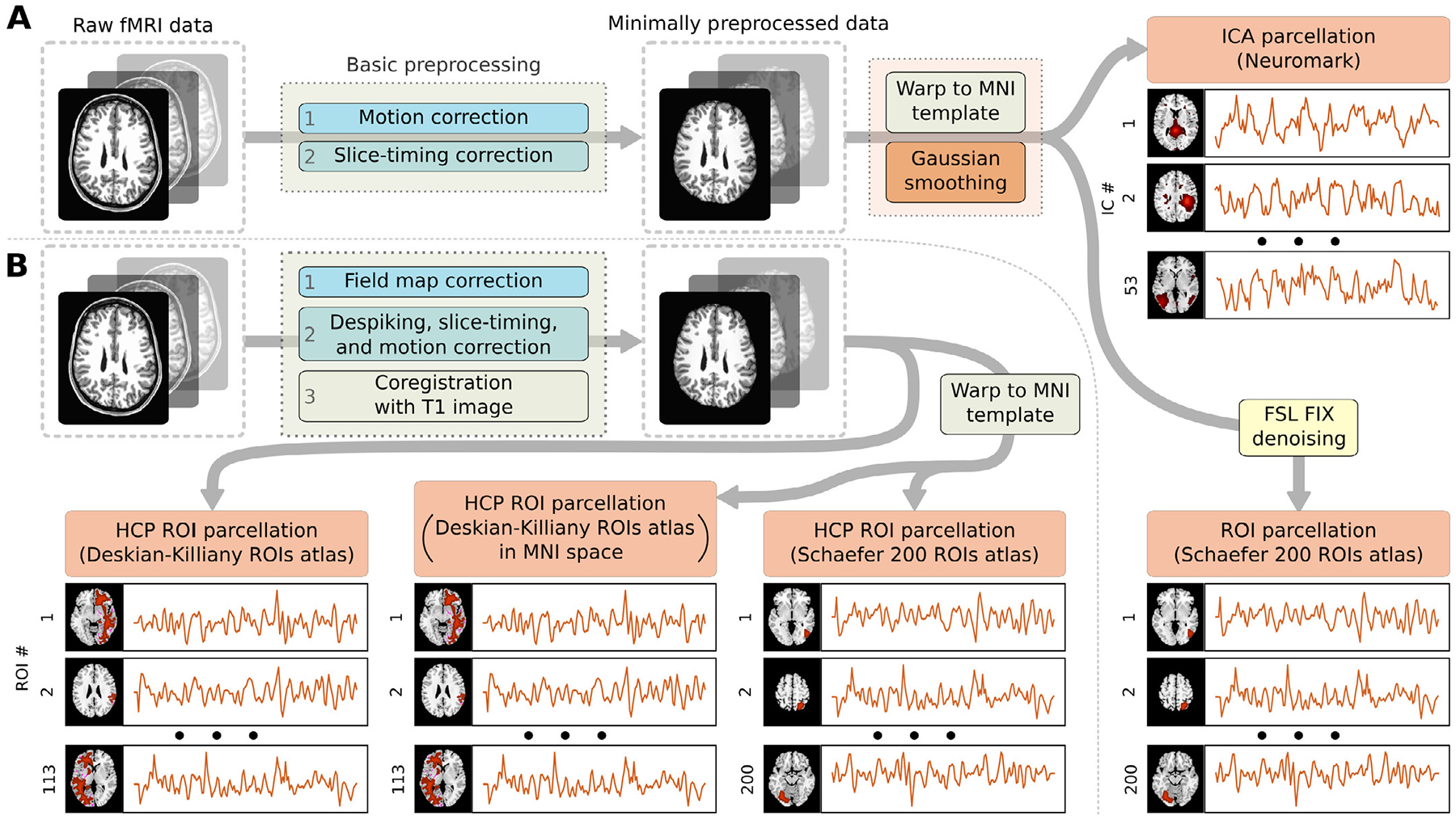
(a) Schematic flowchart of the general fMRI data preprocessing pipeline used to obtain ICA or ROI parcellated data. (b) Schematic flowchart of the additional HCP data preprocessing pipeline.

We further employed two brain parcellation techniques, one based on deriving the regions from the fMRI data using ICA (ICA parcellation), and another based on using regions of interest from a predefined brain atlas for comparison (ROI parcellation). For the ICA parcellation, we used the Neuromark pipeline described by Du et al. (2020) to extract 53 independent components. For the ROI parcellation, we used Schaefer’s 200 regions atlas to extract the average signals from the ROIs; the voxel-level data was preliminarily denoised using FSL’s FIX-ICA technique (Jenkinson et al., 2012). The flowchart of this pre-processing pipeline is shown in Fig. 2(a).

For the UK Biobank dataset on ‘Sex ⊗ Age bins’ category we split the subjects into 10 equally wide 4-years age bins (ages between 30 and 70), and then further split these bins according to subjects’ sex.

For the models expecting the FNC fMRI as their input we computed Pearson correlation matrices from the fMRI time series for each subject.

For the SwiFT model designed for volume fMRI input we used the minimally preprocessed HCP data warped to the MNI space and the minimally preprocessed FBIRN data warped to MNI space and resampled to the HCP data grid. For the models used in comparisons with SwiFT we parcellated the preprocessed data using Schaefer 400 ROIs atlas.

#### 2.2.2. Additional HCP preprocessing pipeline

To analyze the influence of the data preprocessing techniques on the models’ performance we prepared a few differently preprocessed additional HCP datasets. All HCP images were minimally pre-processed using AFNI toolbox (Cox, 1996; Cox and Hyde, 1997), which included field map correction, despiking, motion and slice-timing correction, and coregistration with the T1 images. Then, minimally preprocessed data was warped to an MNI template. The following steps, however, were different across different versions of the dataset. The flowchart on Fig. 2(b) summarizes these additional pipelines.

To assess the influence of the MNI projection, we prepared two versions of the HCP dataset using Desikan-Killiany (DK) brain atlases generated by Freesurfer for each sample in the dataset. In the *original* version of the dataset we extracted the average ROI signals from the minimally pre-processed HCP data in the original subjects space. In the *MNI* version of the dataset we warped the minimally pre-processed HCP data and DK atlases to the MNI template, and only then extracted the ROI signals.

To assess the influence of the brain atlases used for ROI parcellation, we prepared an additional version of the Schaefer 200 ROI HCP dataset to compare it to the DK ROI HCP data. We call this version “noisy” to distinguish it from the Schaefer 200 ROI HCP dataset preprocessed according to the general pipeline. For this dataset we warped the minimally pre-processed HCP data to the MNI template, and then extracted the ROI signals using Schaefer 200 ROI atlas.

In addition to that, in order to analyze the models ability to distinguish the time direction in the data we prepared a special dataset based on HCP ICA data. For this, we took a random half of the samples from the dataset and labeled them as *0* s; then, we took the samples from the other half, flipped them along time axis, and labeled them as *1* s.

### 2.3. Experimental setup

In our experiments we tested how well the models can train on the fMRI datasets, analyzed their performance on differently preprocessed data and data with a reshuffled temporal order, and looked into the models’ spatial attention to reveal the regions important for the accurate classification.

#### 2.3.1. Hyperparameter tuning

Before running the experiments with meanMLP, LSTM, and Transformer models, we needed to find an optimal set of hyperparameters (HPs) for them. For this purpose we singled out a 1∕30th portion of the UKB dataset for the tuning and ran 400 iterations of HP search for each model. In each iteration we randomly sampled a set of HPs, performed stratified 5-fold cross-validated experiments and extracted an average test ROC AUC score. HPs associated with the highest ROC AUC score were chosen as optimal. The tuning portion of the UKB dataset was not used further in the experiments.

#### 2.3.2. Classification performance comparisons

In our work we performed two kinds of classification performance comparisons: one in which we tested the trained models on a test (holdout) data of the training dataset, and another in which we tested them on a different dataset of the same category (information on categories is provided in Table 1).

In order to compare classification performance of the models on fair terms, we performed stratified 5-fold cross-validated (CV) experiments on each dataset with the meanMLP and the rest of the models referenced in Section 2.1. To detect overfitting, we randomly split the training set into the train and validation sets using stratified sampling to preserve class balances. Validation set size was chosen to be 1∕5th of the training data or 16% of the entire dataset, resulting in 64/16/20 train/validation/test splits. We picked the models with the smallest loss on the validation set, tested them on the test set, and computed the test ROC AUC score. For each test fold we repeat the randomized train/val splitting ten times to marginalize over the initialization effects; this way, we obtained 50 test results for each model-dataset pair. We ensured that for each dataset the 50 train/validation/test splits remained consistent across experiments with different models, with no subject’s data being present in two or more of the sets simultaneously.

For the experiments with SwiFT we used the same CV experiment design, but used only one train/validation split for each test fold, resulting in a total of 5 results for a dataset.

Since the fMRI data samples can vary significantly across populations and acquisition sites, it is important for the clinical applications to use models that generalize well on out of sample data. Concerningly, in a recent work by Chekroud et al. (2024) the authors showed that ML models may have poor generalizability on biomedical data. To verify this, in our work we performed what we call “transfer” experiments, in which we tested how well the models, when trained on one dataset, were able to perform classification on another dataset of the same category (FBIRN, BSNIP and COBRE on schizophrenia, and OASIS and ADNI on Alzheimer disease). To do that, in addition to testing the trained models on the test set in the experiments described above we also tested them on the entirety of data from the same-category datasets.

#### 2.3.3. Data analysis experiments

In order to further validate the performance of our meanMLP model we tested it on a differently preprocessed fMRI data, using the HCP datasets described in 2.2.2. However, we also used this opportunity to analyze the influence of different preporcessing techniques on different model architectures. In these experiments we limited our model choice to meanMLP, LSTM, meanLSTM, Transformer, and meanTransformer, as these models’ properties are easier to interpret. Hinted by the meanMLP’s indifference to the temporal order in the data, we additionally tested the performance of these models on the HCP and UKB data with a broken temporal order.

We used the same 5-fold cross-validation experiments design described above. To obtain the data with the broken temporal order, we reshuffled the training set data over the time axis. Each sample from the training set was reshuffled over the time axis independently; on each new training epoch a new shuffling was used. Validation and test sets were left as they are.

#### 2.3.4. Spatial attention

In order to analyze the brain spatial attention of the trained models we employed a gradient-based saliency method (Simonyan et al., 2014), which highlights the features in the input that play an important role for the model’s prediction. Saliency maps were computed for the test set data w.r.t. the true class of an input sample using the 50 models trained as described in 2.3.2. For the models trained on one of the datasets on schizophrenia (FBIRN, BSNIP and COBRE) or Alzheimer disease (OASIS and ADNI), we also computed the saliency maps for the entirety of the data from the remaining same-category datasets. The computed gradients were merged across time points and subjects, grouped according to their true class and compared using Welch’s unequal variances t-test. The model’s spatial attention was then estimated based on the FDR-corrected p-values.

Following the approach described by Lewis et al. (2022), we also computed the temporal pairwise correlations for each saliency map, which we further relate to as co-saliency. By grouping the co-saliencies according to their true class, we performed Welch’s unequal variances t-test on the co-saliency groups to reveal statistically significant differences in the model’s attention for one class or another.

## 3. Results

In this section we present the results of our experiments. We (i) evaluate the classification performance of our model and its counterparts on fMRI datasets in terms of accuracy and training time, (ii) analyze the models’ performance on the data with reshuffled temporal order and on a differently preporcessed data, and (iii) introspect the trained meanMLP model by visualizing its predictions over time and computing saliency maps.

### 3.1. Classification comparisons

#### General comparisons.

**Fig. 3.**
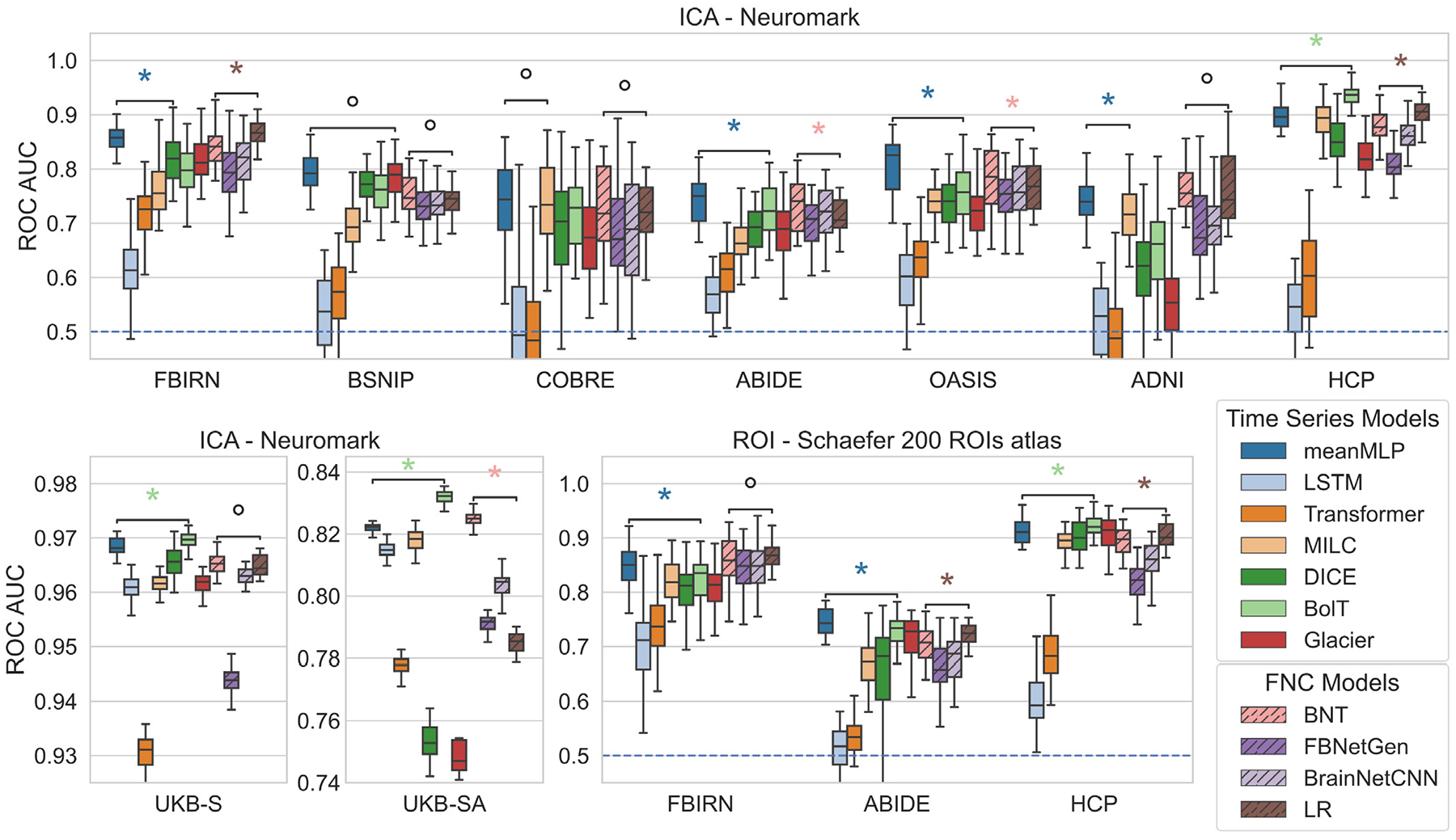
Comparison of test ROC AUC scores for the meanMLP model and other considered models on various datasets in a classification task. The meanMLP model shows competitive results compared to more advanced models for fMRI time series, as does logistic regression (LR) trained on FNC data. The blue dashed line at ROC AUC = 0.5 denotes a random choice baseline. The asterisk and degree signs denote significant (*p* < 0.05) and insignificant (*p* > 0.05) statistical differences between model results according to the Wilcoxon rank test. We ran these tests on meanMLP and the next best TS model, and LR and the next best FNC model.

**Fig. 4.**
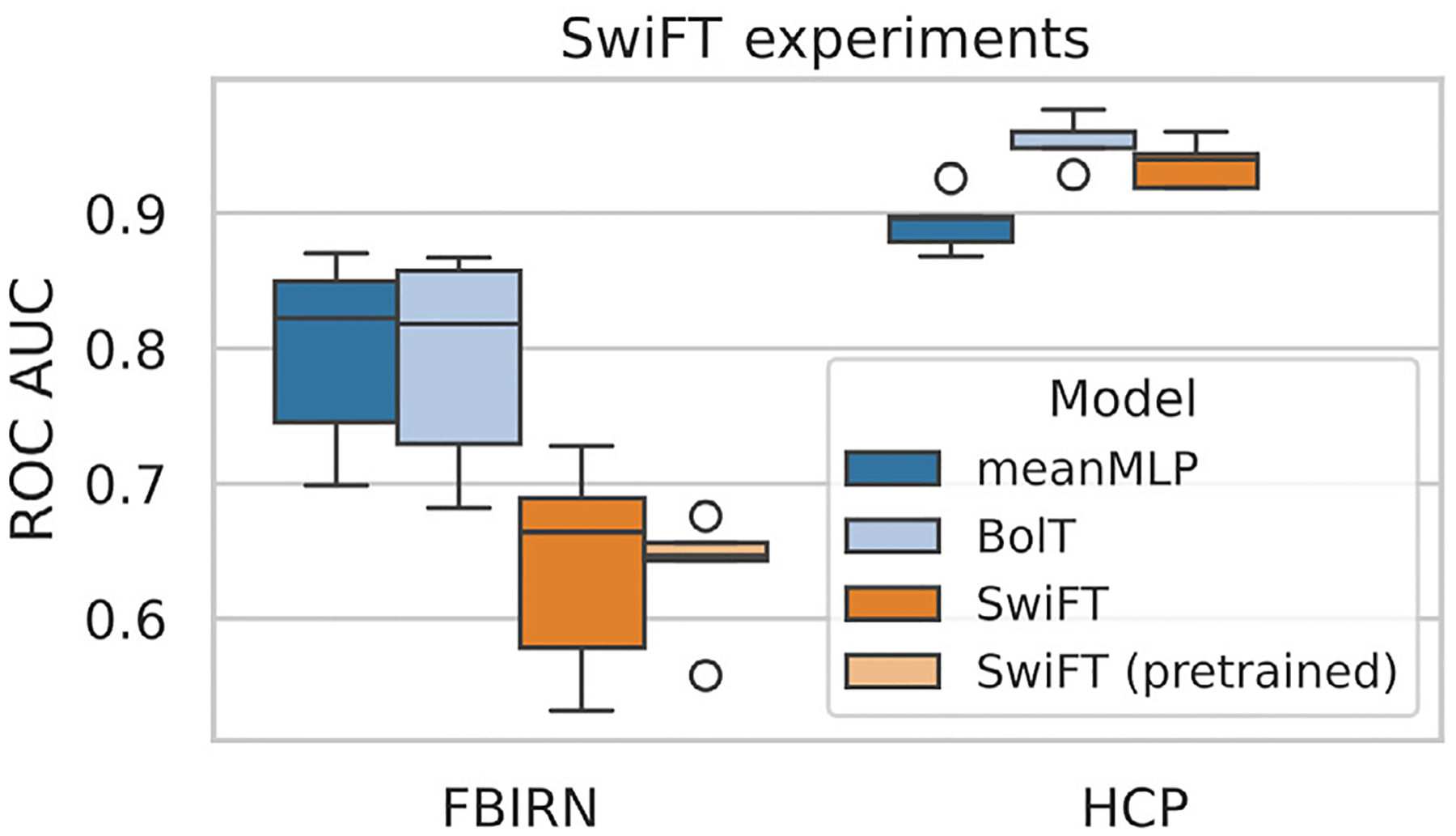
Comparison of test ROC AUC scores of meanMLP, BolT, and SwiFT models on the FBIRN and HCP datasets. In these experiments, we used 5-fold CV with one trial for each fold, unlike the experiments in Fig. 3 where we ran 10 trials. SwiFT was trained on volume fMRI time series, while meanMLP and BolT models were trained on parcellated fMRI time series using the Schaefer 400 ROI atlas, which is native to the BolT model. The results we can see here conform with the observations from Fig. 3 — meanMLP model shows better results on the smaller datasets (like FBIRN), but falls behind more intricate models when more data is available.

Using the experimental setup described in Section 2.3.2, we trained our models for classification on different datasets. Fig. 3 shows the test ROC AUC scores of the trained models. As we can see, the meanMLP model performs on a competitive level with other, more advanced models on various tasks, often showing the best results across time series models. LR exhibits a similar behavior, showing competitive results among the FNC models, although in a less pronounced way compared to the meanMLP. This behavior is observed on different classification tasks and different fMRI parcellations. In the case of multiclass classification (UKB-SA) LR falls behind the more intricate models; meanMLP, however, still shows decent results. Notably, on larger datasets (UKB, HCP), meanMLP starts to lag behind the more sophisticated BolT model. We observe a similar behavior in the experiments comparing meanMLP to SwiFT, the results of which are shown in Fig. 4.

#### Training time.

Table 2 displays the relative training times of the models. Here, the meanMLP and LR models excel, consistently demonstrating the fastest performance across the time series and FNC models, respectively, owing to their simplicity.

#### Transfer comparisons.

**Fig. 5.**
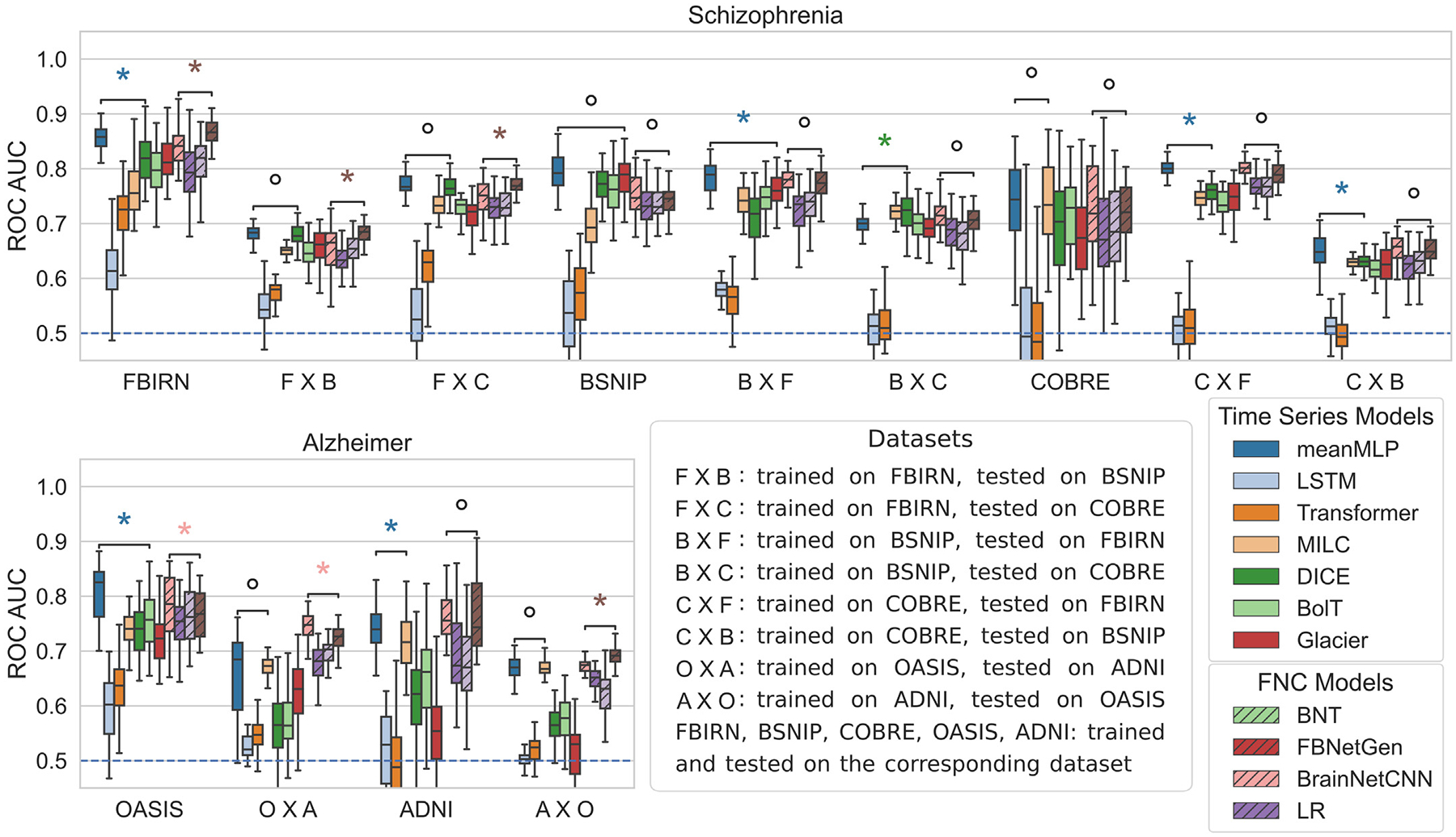
Comparison of test ROC AUC scores of the meanMLP model and other considered models when trained on one dataset and tested on another. All of the datasets here are ICA datasets. The meanMLP model again shows competitive results compared to more advanced models for fMRI time series, as does logistic regression (LR) trained on FNC data. The blue dashed line at ROC AUC = 0.5 denotes a random choice baseline. The asterisk and degree signs denote significant (*p* < 0.05) and insignificant (*p* > 0.05) statistical differences between model results according to the Wilcoxon rank test. We ran these tests on meanMLP and the next best TS model, and LR and the next best FNC model.

In order to better assess models’ generalizability, in addition to same-dataset testing we also explored how well the trained models “transfer” on the datasets of the same category (FBIRN, COBRE, and BSNIP on schizophrenia, OASIS and ADNI on Alzheimer disease). Fig. 5 show the results of these tests. The meanMLP model again performs on a competitive level with more advanced time series models even when applied to a data from a different dataset. Although, as we see, this time the complexity of other models sometimes allows them to generalize better. LR exhibits a similar behavior, showing competitive results among the FNC models.

This result is especially interesting in the light of a recent paper by Chekroud et al. (2024), where machine learning models, when trained on a biomedical data to distinguish the clinical output of schizophrenia treatment, were shown to fail on the independently collected data. Our results show that, when it comes to fMRI data, the machine learning models are quite capable of transferring their performance on the independent data.

### 3.2. Time-shuffled training

In pursuit to understand the reasons behind the meanMLP’s classification success we decided to explore the importance of the temporal order in fMRI time series for the accurate classification. Why temporal order? As was noted in Section 2.1.1, the meanMLP architecture lacks remarkable features with the exception of being indifferent to the temporal order in the data. This fact is supposed to harm the meanMLP ability to perform classification on time series, unless the time points in the time series, when considered in isolation from each other, contain enough discriminative information.

**Fig. 6.**
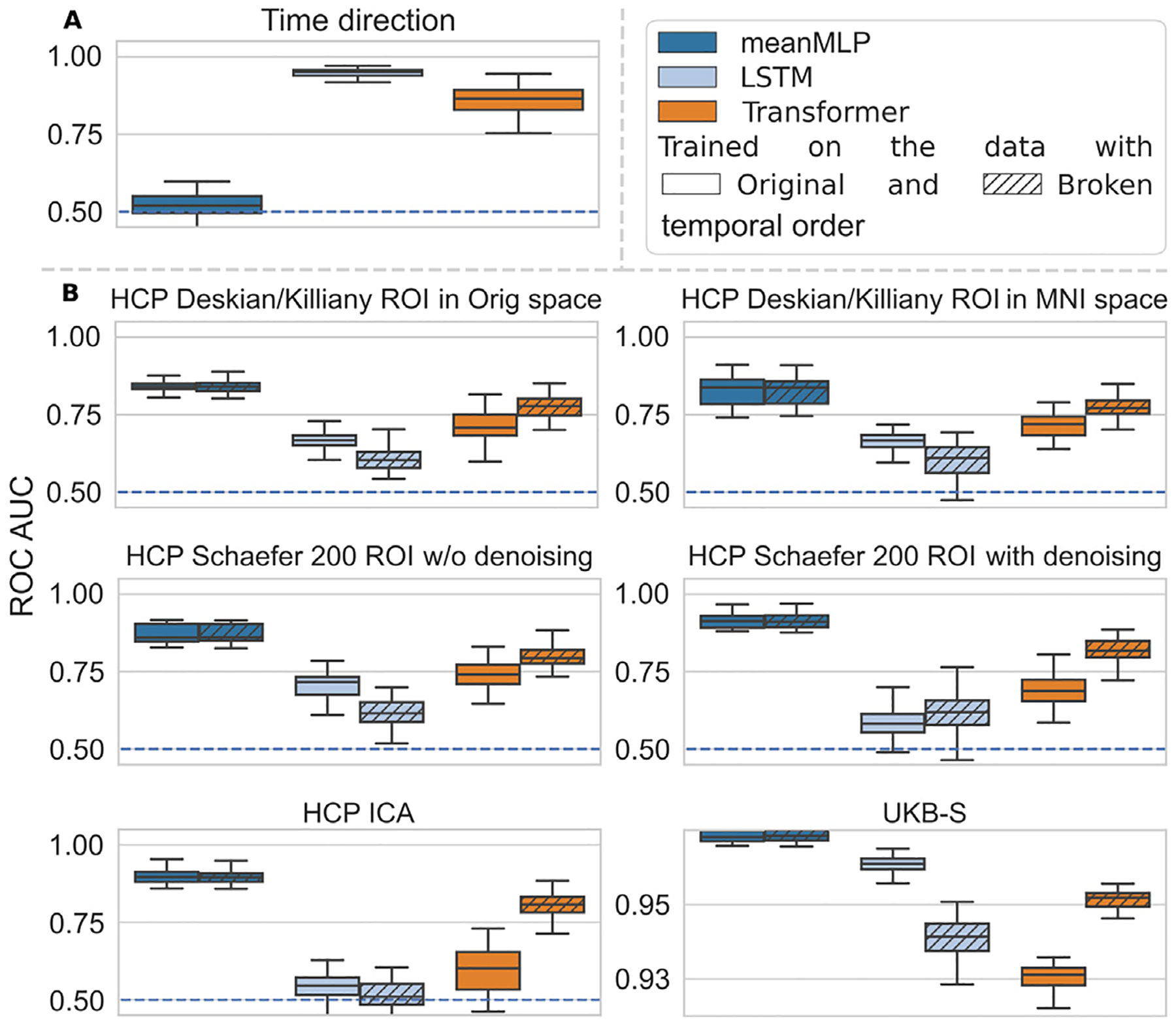
Comparisons of classification performance of a few test models trained to distinguish (a) the time direction in the HCP ICA data, and (b) the subject sex in the HCP and UKB data with original and broken time order. (a) We trained our test models for the time direction inference task using the special relabeled HCP ICA data described in Section 2.3.3. LSTM and Transformer are capable of solving this problem to some extent, showing a general ability to learn sequential features. meanMLP fails at it completely, as can be expected from the model’s architectures. (b) To break the temporal order in the data we reshuffled the samples from the training set along the time direction on every training epoch. meanMLP’s performance was not affected by the broken temporal order. Interestingly, order-aware LSTM and Transformer models managed to train even on the data devoid of sequential features, which is prominently seen on the UKB-S results.

To investigate this, we selected a narrow pool of models with well known capability for processing the sequential data, namely LSTM and Transformer, and trained them on two tasks along with meanMLP. In the first task we train the selected models to distinguish the fMRI samples with normal and inverted temporal order, using the special HCP ICA dataset described in Section 2.3.3. The intention behind this task is, on the one hand, to verify the existence of sequential features hidden in the fMRI temporal order, and, on the other hand, to analyze the models’ ability to detect these features and use them in classification. The results of the models on this task are shown in Fig. 6(a). We can see that LSTM and Transformer models are able to detect some sequential features and thus distinguish the regular and time-reversed fMRI samples. meanMLP fails at this task, as expected, due to its architecture.

In the second task we trained the selected models on the HCP and UKB data, in which we artificially broke the temporal order. We did so by randomly reshuffling each training data sample along the time axis. Each sample was reshuffled independently from the others, and was reshuffled anew on each new epoch. Such reshuffling is supposed to put any sequential features in the data in disarray and force the models to look for some stationary discriminative features, which are independent from the temporal order. We know that such features exist, since the meanMLP model cannot use anything else for classification. So, this task allows us to explore how the performance of order-aware models changes when they are left with only stationary fMRI features. In this task we used a few variations of HCP dataset described in Sections 2.2 and 2.2.2 in order to rule out the influence of preprocessing techniques on the temporal and stationary fMRI features, at least to some extent, and the UKB-S dataset, on which all of the considered models clearly proved their classification capabilities.

Fig. 6(b) shows the results of models on the second task. As expected, meanMLP is indifferent to the temporal shuffling. Overall, all of the considered models are able to train on the data with the broken temporal order, which is best shown by the results on the UKB-S dataset. More intriguingly, the Transformer model tends to benefit from the broken temporal order, sometimes significantly. The LSTM model performance slightly degrades on the data with broken temporal order with an exception of a single dataset.

We also conducted this kind of experiment with the rest of TS models, the results of which are shown in Appendix.

### 3.3. Influence of preprocessing

In order to verify the meanMLP results on one hand, and to further compare the behavior of order-aware and order-indifferent architectures on another hand, we compare the performance of a few chosen models on the differently pre-processed fMRI data. Here we consider the same pool of models (meanMLP, LSTM, and Transformer) as in the previous section, with the addition of meanLSTM and meanTransformer models. We use the HCP datasets described in Sections 2.2 and 2.2.2, which allow us to compare the influence of a few preprocessing techniques in an isolated environment.

**Fig. 7.**
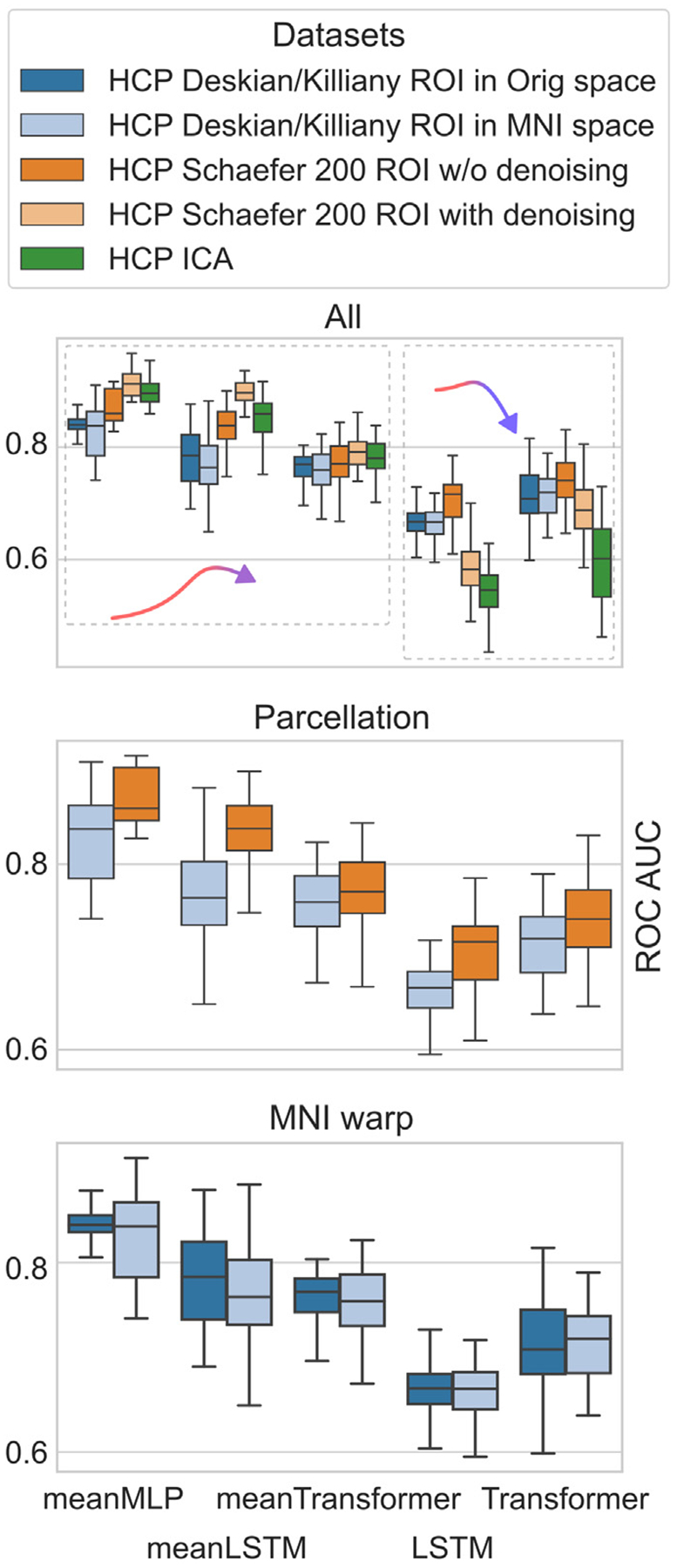
Influence of the data preprocessing techniques on models classification performance. While different preprocessing techniques can improve the performance of some models and hurt the others, the models ranking remains mostly unaffected. Judging by the performance comparisons on all the available data, we can distinguish two categories of models: mean (meanMLP, meanLSTM, and meanTransformer) and regular (LSTM and Transformer). While mean models tend to perform better on the HCP data that was prepared according general preprocessing pipeline (last two datasets) compared to the additional pipeline (first three datasets), regular models do the opposite. At the same time, the use of different brain atlases and the normalization to the MNI space do not show such effect.

Fig. 7 shows the results of our comparisons. We can see that the warp to MNI space does not significantly affect the models’ classification abilities, and the use of different brain atlases for fMRI parcellation, while affecting the models performance, affects it in the same way. However, we notice the differences in performance on the HCP data prepared according to the general and the additional preprocessing pipelines. Based on these differences, we can distinguish two groups of models: one group includes LSTM and Transformer, two order-aware models that perform better on the HCP data prepared according to the general pipeline; the other group consists of Mean models that perform better on the HCP data prepared according to the additional pipeline.

More importantly, we can see that while pre-processing affects the models’ performance, it does not change the models’ ranking significantly. meanMLP shows best results across the chosen pool models. Interestingly, the introduction of the averaging step to LSTM and Transformer models improved their performance significantly, as we can see from the meanLSTM and meanTransformer results.

### 3.4. Introspection into the prediction dynamics

**Fig. 8.**
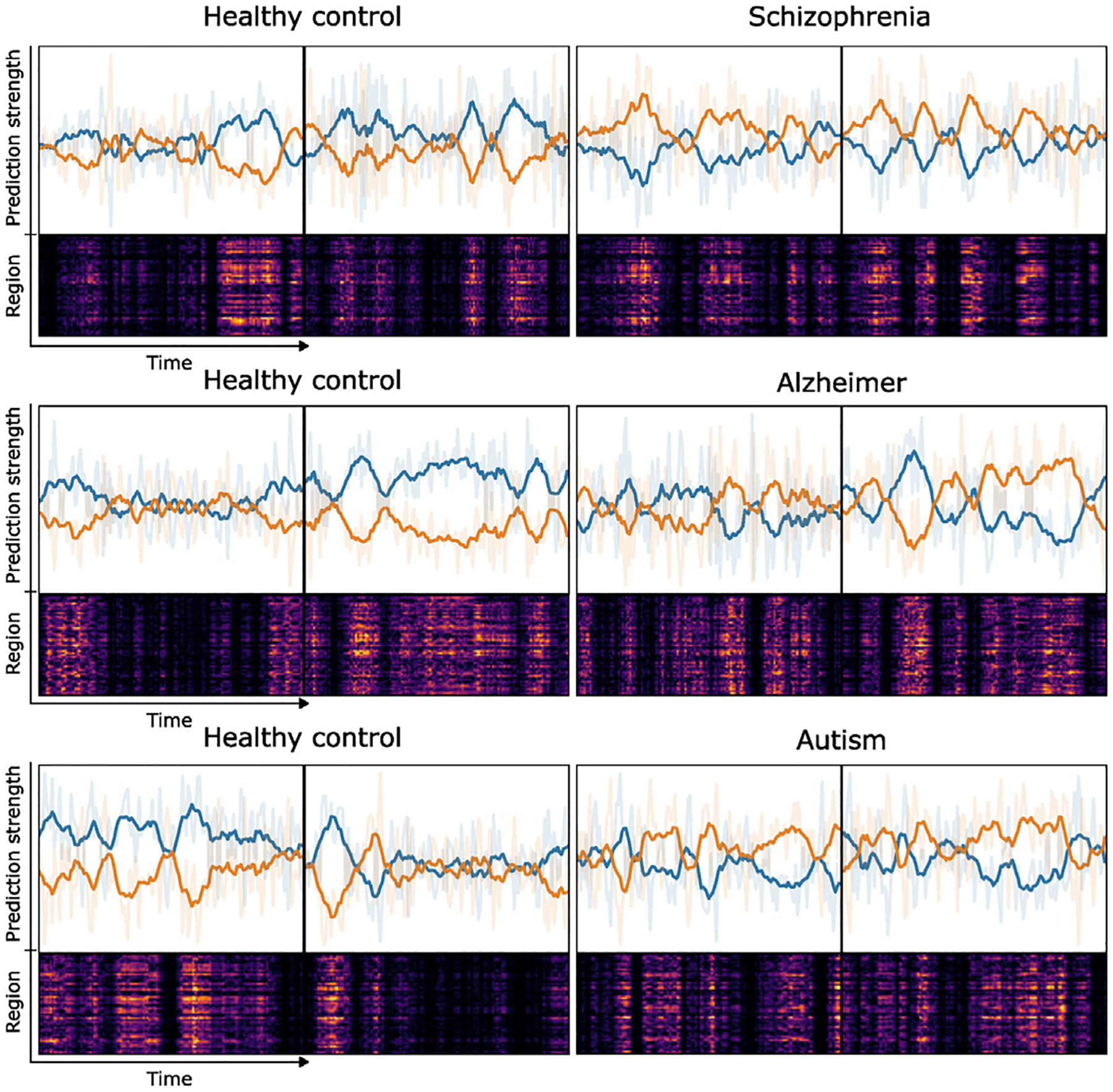
Dynamics in MLP block predictions in classification of schizophrenia (FBIRN), Alzheimer disease (OASIS), and autism (ABIDE). Two healthy control subjects and two subjects with brain disorder were taken from each respective dataset. The upper part of the plots shows the relative prediction strength for each class at each time point. Solid color lines visualize the predictions averaged over the 10 time points window. Ghost color lines in the background visualize the raw predictions. The lower part of the plots show the saliency map, computed using integrated gradients (Sundararajan et al., 2017) for the true label’s output logit and multiplied at each time point by the corresponding prediction strength. The existence of time frames where the prediction strength for one class persistently exceeds the other suggests the presence of periods of anomalous and exclusively normal brain activity.

Although meanMLP model is capable of accurate classification of the brain disorders without learning any dynamical information from the data, it does not mean that this information is not there, and it is still possible to use this model to peek into the dynamics by inspecting the output logits of the MLP block before the averaging step. Fig. 8 shows the results of introspection into the MLP block predictions. As we see, there are periods of time where the prediction strength for one class consistently exceeds the prediction strength for the other, which suggest the existence of normal and abnormal brain activity over periods of time. This fact indirectly verifies the existence of dynamics in the data. However, as previous results show, the knowledge of these dynamics is not necessary for the accurate classification of brain disorders.

### 3.5. Spatial attention

#### Region attention.

Using the meanMLP model trained on BSNIP dataset, we explored what brain regions the model found to be most discriminative for the classification of schizophrenia. To do that we computed the saliency maps and found regions for which the gradients computed on the data of different classes were significantly different according to Welch’s t-test statistics, as described in 2.3.4.

**Fig. 9.**
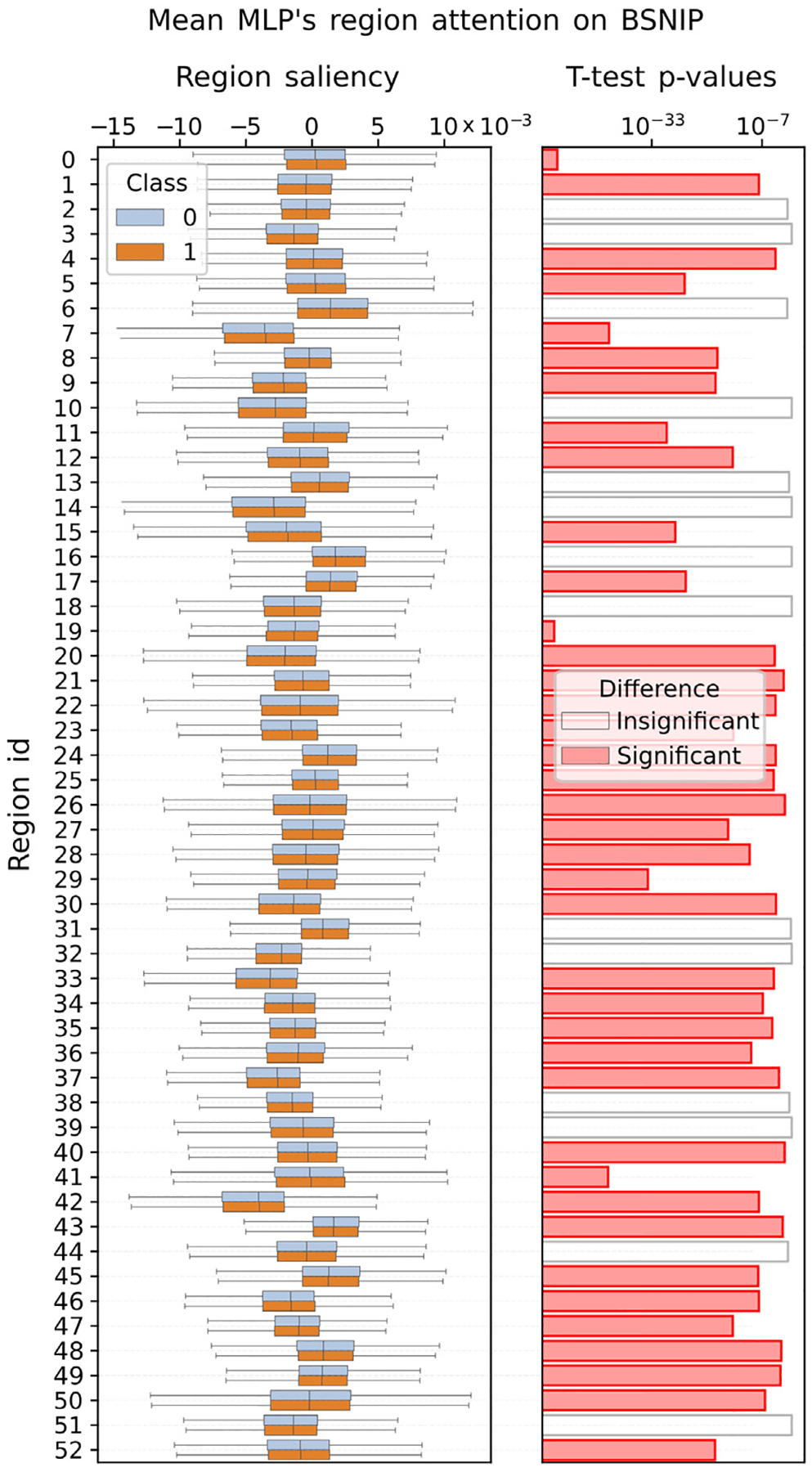
Per-region comparisons of gradients computed on test BSNIP data using meanMLP model trained on BSNIP. The left panel shows the distribution of gradients computed for the samples of each class at each region; the right panel shows FDR-corrected p-values of the Welch’s t-test applied to the gradient distributions. The significance of region differences was determined by p-values < 0.05. While the perregion distributions of gradients on the left panel appear to be fairly similar to each other, the p-values from the Welch’s t-test indicate that most of the region distributions are significantly different, which makes the interpretation of the model’s attention overly complex.

Fig. 9 shows the results of these comparisons. As we see, the gradients from a variety of regions turned out to be statistically significantly different. This fact makes it difficult to draw conclusions about the importance of individual brain regions for the schizophrenia classification, as too many of them appear to be important to the model. In a way, this failed attempt to interpret the model signifies a different kind of importance — the importance of designing more interpretable models for neuroimaging data, if we hope to learn the mechanisms of brain work through the machine learning. A similar analysis was performed on datasets other than BSNIP; its conclusions, however, were the same.

#### Correlational attention.

**Fig. 10.**
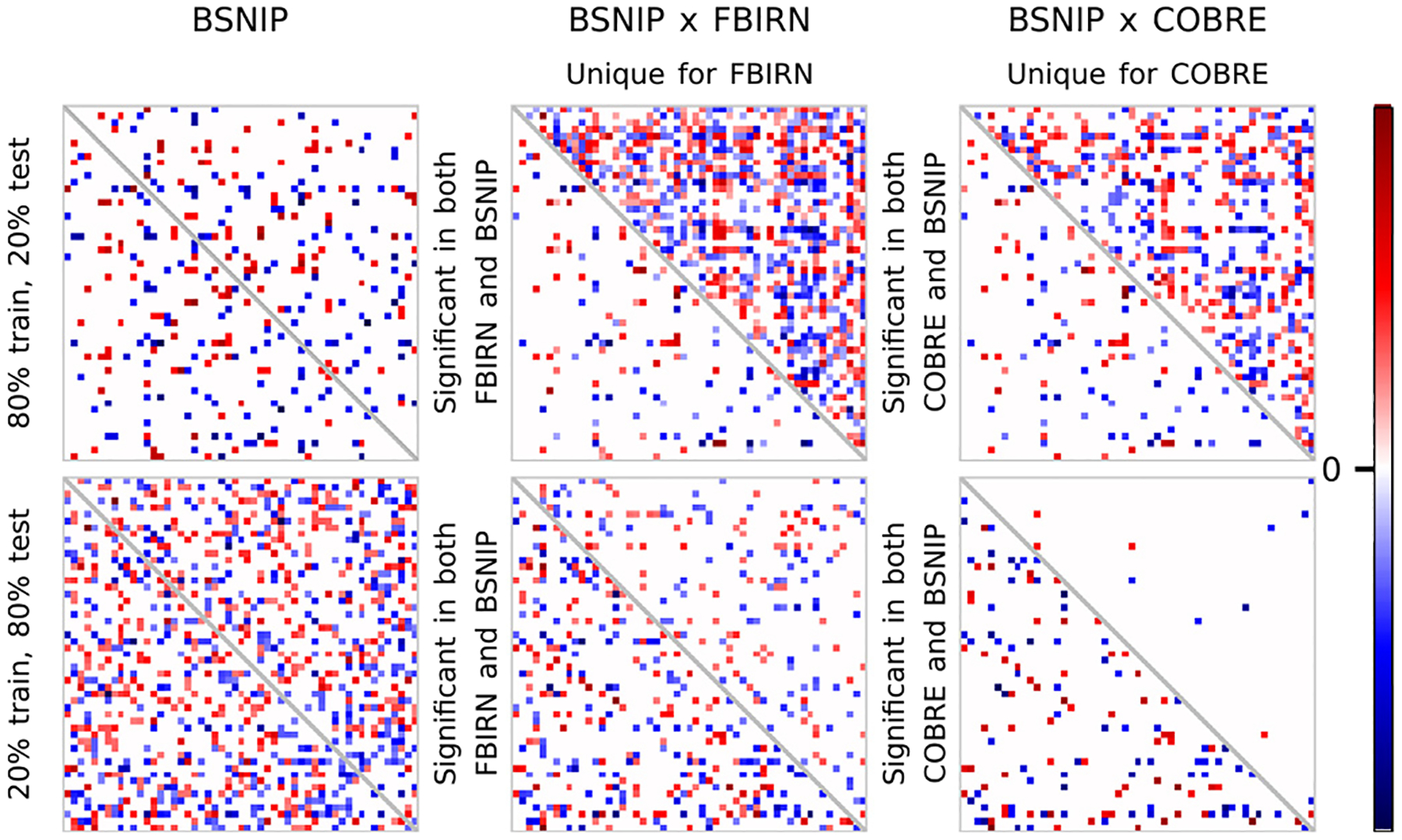
Comparison of co-saliencies computed from meanMLP trained on BSNIP. We show t-values of Welch’s t-test applied to group co-saliencies computed from the test BSNIP, the entire FBIRN, and the entire COBRE datasets. Only the significant t-values with the corresponding p-values < 0.05 are shown on the panels. The FBIRN and BSNIP panels are split into 2 parts along the main diagonal: the lower triangles show components that are significant in both COBRE and BSNIP data, the upper triangles show the components that are unique for COBRE. The first row shows the results for the meanMLP model trained on the BSNIP data with 80% training and 20% test splits, as described in 2.3.2, while the second row shows the results for which this proportion was flipped to 20% training and 80% test.

With the previous result being a failure, we tried a different approach to the attention problem by statistically comparing not region saliencies, but rather the temporal correlations of regions saliencies that we call co-saliencies. The results of this approach for the meanMLP trained on BSNIP are shown in Fig. 10. Here we also considered the co-saliencies computed from the FBIRN and COBRE data using the same meanMLP trained on BSNIP in order to better understand how the model transfers onto other datasets.

Judging by the BSNIP panel in the first row, we can see that this approach does not provide us any interpretable spatial attention either, as too many components in co-saliencies are significantly different between classes, and they also do not appear to be clustered. However, the salient components on the transfer dataset panels indicate that while the model detects some familiar features in the transfer data (regions in lower triangles), it also pays attention to some new regions that were not salient in the training dataset (regions in upper triangles). This effect may lead to intriguing interpretations regarding the learning process of supervised learning.

However, it is not clear how much this observation is affected by the size effect of the data. The second row of results in Fig. 10 shows the salient regions of a model for which the proportions of train and test sets of the BSNIP dataset were flipped. From these results we can see that the model trained on less data pays attention to more regions in BSNIP data; yet it pays attention to less regions in the transfer datasets. We reserve the comprehensive explanation of these observations for the future work.

## 4. Discussion

In our experiments we found that the proposed meanMLP model is a surprisingly decent classifier for the fMRI data, and also revealed an interesting property of the fMRI data itself. We believe these findings carry important implications to the researchers working on joint ML/neuroimaging projects, especially the work involving the fMRI data.

### 4.1. Implications to the fMRI classification accuracy as a models evaluation metric

Our experimental findings in Section 3.1 indicate that the meanMLP model can successfully classify mental disorders, sex, and age based on the fMRI time series. Notably, meanMLP performance is competitive to that of the best models for fMRI time series data classification, despite much simpler model design, and only falls behind the more intricate models when more data is available. This conclusion holds on differently pre-processed fMRI data, as shown in Section 3.3.

We believe that the above fact, combined with the success of logistic regression on the FNC fMRI data, underscores the necessity of reassessing the motivation for the future research on ML applications to brain fMRI data. While achieving a decent classification accuracy remains an important problem for the real world medical applications, our findings reveal that the state-of-the-art accuracy can be achieved with relatively simple methods. At the same time, the increasing model complexity often leads to either none or disproportionately small accuracy improvement on most of the tasks. Thus, it appears more fruitful to explore the ML applications to other neuroscience challenges, such as fMRI data explainability, where greater model complexity can allow us to delve deeper into the intricacies of brain function.

### 4.2. Importance of fMRI dynamic information for classification

We believe that the classification success of the meanMLP model on one hand, and the classification results of the models trained on the fMRI data with a broken temporal order on the other hand provide us an intriguing insight on the fMRI dynamics. A discriminative information in the fMRI time series can be potentially embedded by two kinds of features: dynamical *sequential features*, hidden in the fMRI temporal order, and *stationary features*, independent from the temporal order. meanMLP is incapable of learning the sequential features, as it is insensitive to the temporal order in the data by design; yet it still shows decent classification results by using only stationary features. In the experiments with the broken temporal order, where the sequential features are artificially degraded, a few chosen order-aware models are still able to learn to perform the classification task, presumably using only stationary features. Notably, their performance does not degrade as significantly as could be expected; on the contrary, the Transformer model even improves its performance on the data with the broken temporal order. In this latter case the broken temporal orders probably plays a role of regularization through data augmentation.

These results collectively provide evidence that *the sequential features—and, by extension, the fMRI dynamics—may contain significantly less discriminative information for fMRI classification problems than is commonly believed*. Another plausible explanation for these observations is that sequential features may be inherently more difficult to detect than stationary ones, and the order-aware models used in our experiments are simply unable to learn these features effectively.

We do observe one exception: the BolT and SwiFT models outperform the meanMLP when trained on the HCP and UKB datasets, two of the larger datasets. Since these models are among the largest architectures we tested, this could be an instance of scaling laws at work (Abrol et al., 2021). It is also possible that the hierarchical structure of BolT and SwiFT allows them to learn discriminative sequential features more efficiently than other models when sufficient data is available, enabling these models to outperform meanMLP on this task.

It is worth noting that the relative importance of the sequential and stationary features depends on the specific task at hand. As such, while meanMLP exhibits a decent performance in classifying brain disorders, it is unable to distinguish the time direction in the fMRI data, which is a purely *temporal* task. We believe that this fact can be exploited in the future research to gain deeper insights into the dynamic aspects of the phenomena underlying the classification task. For instance, if, in a given task, the sequential features prove to be more important than stationary ones, this would imply that the phenomena behind the task manifest itself more in fMRI dynamics. Such analysis, however, requires more reliable methods for detection of sequential features, as the indirect method based on temporal shuffling we used in our work can only destroy such features. Perhaps the dynamical systems theory (John et al., 2022) can provide such methods.

Additionally, these observations carry profound implications for research aimed at enhancing the fMRI data explainability. Researchers who may employ ML models to uncover sequential features and use the classification performance as a validation metric should be aware that these models may, in fact, unveil stationary features instead. This awareness may be critical for ensuring the accurate interpretation of model outcomes.

Finally, in our work we considered only the resting-state fMRI data. Whether the task-based fMRI data exhibits the same properties remains unclear.

## 5. Conclusions

In our work, we present the meanMLP, a simplistic model designed for the classification of sequence data, particularly in the context of resting-state fMRI analysis. Through extensive comparisons, we show that meanMLP is capable of classification of brain disorders, sex, and age from the resting-state fMRI with remarkable accuracy. We hence propose our model as a baseline for future models for fMRI time series classification.

Given the effectiveness of both our model and logistic regression on FNC fMRI data, we advocate for a shift in focus toward exploring problems beyond classification accuracy in future joint neuroimaging/ML research. While current trends often emphasize increasingly complex models in pursuit of higher classification performance, our findings reveal that simpler models are quite capable of achieving comparable and even surpassing results on the fMRI data. This complexity, however, may be better suited for addressing other challenges, such as enhancing neuroimaging data explainability.

In support of this idea, we attempted to use our model’s spatial attention to reveal the discriminative features of the fMRI data. However, our efforts were unsuccessful, as we found too many statistically significant differences between the explanations the model generated for different groups. Nonetheless, our exploration of the model’s insensitivity to temporal order revealed a more intriguing characteristic of fMRI time series. Contrary to intuitive assumptions, our experiments suggest that the temporal order of fMRI data may contain much less discriminative information than usually believed. This finding, corroborated by experiments with temporally re-shuffled data, underscores the need to consider the role of fMRI dynamics more critically in future research aiming to uncover meaningful features in the data using machine learning techniques.

## Figures and Tables

**Table 1:** Information on the datasets used in the experiments.

Dataset	Category	Parcellation	Subjects	Time length	# classes
FBIRN (Keator et al., 2016)	Schizophrenia	ICA	311	140	2
COBRE (Çetin et al., 2014)	Schizophrenia	ICA	157	140	2
BSNIP (Tamminga et al., 2014)	Schizophrenia	ICA	589	230	2
ABIDE (Di Martino et al., 2014)	Autism	ICA	869	295	2
OASIS (Rubin et al., 1998)	Alzheimer	ICA	823	156	2
ADNI (Petersen et al., 2010)	Alzheimer	ICA	499	194	2
HCP (Van Essen et al., 2013)	Sex	ICA	833	1185	2
UK Biobank (UKB-S)	Sex	ICA	35 852	490	2
UK Biobank (UKB-SA)	Sex⊗Age bins	ICA	35 852	490	20
FBIRN	Schizophrenia	Schaefer 200	311	160	2
ABIDE	Autism	Schaefer 200	871	316	2
HCP	Sex	Schaefer 200	752	1200	2

ICA parcellated datasets have 53 features at each time point; Schaefer 200 ROI parcellated datasets have 200 features.

**Table 2:** Relative training time of the considered models on different tasks.

Datasets	Models								
	meanMLP	Transformer	LSTM	DICE	MILC	BrainNetCNN	FBNetGen	BNT	LR
FBIRN	1	2.3×	3×	4×	13×	163×	95×	66×	1
BSNIP	1	3×	4×	7×	15×	146×	100×	55×	1
COBRE	1	1.6×	5×	5×	28×	168×	109×	95×	1
ABIDE	1	8×	6×	13×	28×	155×	145×	52×	1
OASIS	1	5×	6×	9×	21×	170×	121×	59×	1
ADNI	1	5×	5×	10×	31×	159×	106×	68×	1
HCP	1	12×	5×	13×	35×	171×	412×	58×	1
UK Biobank (Sex)	1	8×	6×	13×	–	102×	57×	24×	1
UK Biobank (Age-Sex)	1	10×	11×	15×	–	67×	34×	14×	1
FBIRN_ROI_	1	2.1×	2.8×	46×	11×	63×	10×	6×	1
ABIDE_ROI_	1	4×	2.5×	79×	14×	19×	5×	1.7×	1
HCP_ROI_	1	2.6×	1.9×	45×	5×	58×	44×	5×	1

All models left to BrainNetCNN are time series models; all models right to MILC are FNC models. Time series models’ times are normalized to the time of meanMLP; FNC models are normalized to LR. We use the average training time across 50 runs. Datasets without specified parcellation are ICA datasets. The MILC model entries on the UKB datasets are missing, since the MILC model was trained on a different hardware on these datasets. BolT, Glacier, and SwiFT entries are missing for the same reason.

**Fig. 11.**
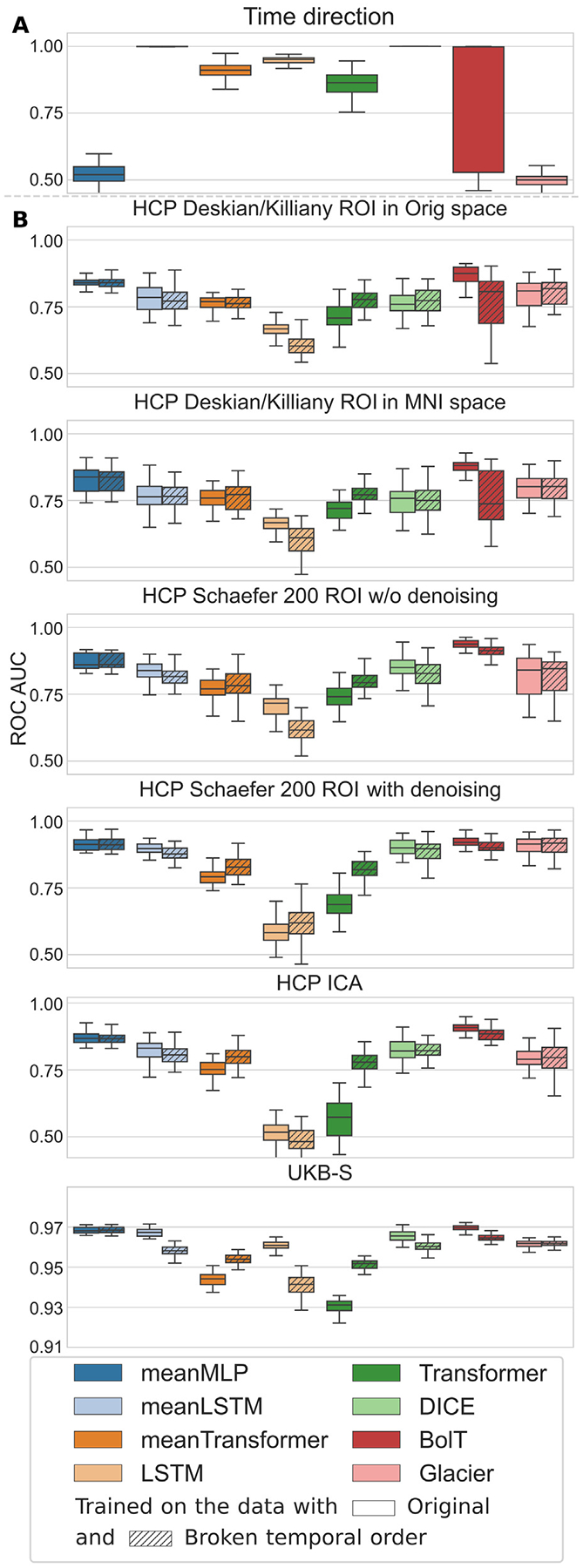
Test classification performance comparison of a wider pool of TS models trained to distinguish (a) the time direction in the HCP ICA data, and (b) the subject sex in the HCP and UKB data with original and broken time order.

## Data Availability

The model implementations and the experimental setup used in our work can be found at https://github.com/neuroneural/meanMLP. This work does not introduce any new datasets; all datasets used in our work are properly referenced in the body of the paper.

## Footnotes

1 https://biobank.ndph.ox.ac.uk/ukb/.

2 http://www.fil.ion.ucl.ac.uk/spm/.

## Appendix. More time-shuffled training

Here we show the results of a wider pool of models trained to (i) distinguish the direction of time in the input, and (ii) trained on the HCP and UKB reshuffled time series. The results of these experiments are shown in Fig. 11. Here we notice an interesting behavior of BolT and Glacier models. Glacier appears to be unable to distinguish the time direction in the fMRI data, and it is not significantly affected by time shuffling, which suggests that it is not actually learning any sequential features. BolT is able to distinguish time directions well, but only in around half of the experiments. Unlike vanilla transformers, its performance is degraded by time shuffling. meanLSTM and meanTransformer behave similarly to their regular counterparts, although they consistently show better classification scores.
